# Who pays for surgical care in the global south? A narrative review

**DOI:** 10.1371/journal.pgph.0004781

**Published:** 2025-06-17

**Authors:** Sanjay Kumar Yadav, Aman Bakhsh, Dhananjaya Sharma

**Affiliations:** 1 Department of Surgery, Breast and Endocrine Surgery Unit, NSCB Medical College, Jabalpur, India; 2 NSCB Medical College, Jabalpur, India; 3 Department of Surgery, NSCB Medical College, Jabalpur, India; Royal Infirmary of Edinburgh, UNITED KINGDOM OF GREAT BRITAIN AND NORTHERN IRELAND

## Abstract

For decades, surgical care was sidelined in global health policy, perceived as costly, complex, and secondary to communicable disease control. However, the past two decades have witnessed a paradigm shift, with evidence highlighting surgery’s critical role in addressing nearly 30% of the global disease burden. Landmark efforts like the 2015 Lancet Commission on Global Surgery and WHO Resolution WHA68.15 underscored that safe, timely, and affordable surgical care is indispensable to achieving Universal Health Coverage and the Sustainable Development Goals. Yet, despite increasing advocacy, a fundamental question remains inadequately addressed: who pays for surgical care in the Global South? This narrative review explores the current landscape of surgical financing in low- and middle-income countries, examining domestic public funding, insurance-based models, donor assistance, diaspora contributions, and the persistent burden of out-of-pocket payments. While some initiatives—such as India’s AB PM-JAY and organizations like Smile Train and KidsOR—illustrate scalable financing models, most low- and middle-income countries still rely on fragmented, underfunded systems that lead to catastrophic health expenditures. Moreover, political neglect, lack of standardized surgical metrics, and low visibility within global health frameworks continue to hinder sustained investment. The review further highlights the cost-effectiveness and economic benefits of surgical interventions, positioning surgery not only as a clinical imperative but also as a strategic investment in national development. Emphasizing the emerging concept of value-based surgery, it argues for integrating frugal innovations and systems-based approaches into health financing frameworks. To achieve equitable and sustainable surgical systems in the Global South, the global health community must address the multifaceted barriers to financing—economic, structural, and political. The review calls for strategic investments, better data, and policy integration to ensure that surgical care is no longer a privilege but a universal right.

## Introduction

For much of the 20th century, surgery was perceived as too expensive, too complex, and not cost-effective in resource-limited settings. Consequently, it was largely omitted from global health funding priorities and development programs and was considered the neglected stepchild of global health. The dominant focus remained on infectious diseases and maternal-child health interventions, seen as more straightforward and scalable. A turning point began in the early 2000s, as voices emerged advocating for the inclusion of surgery in essential health services [1,2]. Although surgical conditions contribute to approximately 30% of the global disease burden, access to safe and affordable surgical care remained severely limited in resource-constrained settings [3]. *The 2015 Lancet Commission on Global Surgery* sounded a clarion call, asserting that five billion people lack access to safe, timely, and affordable surgical care, and that universal health coverage cannot be achieved without investing in surgical services [4]. It pointed out that global funding mechanisms for health—traditionally centered on communicable diseases such as HIV/AIDS, tuberculosis, and malaria—had not adequately extended to surgical care. Each year, an estimated 16.9 million deaths are attributed to conditions requiring surgical intervention—surpassing the combined annual deaths from HIV/AIDS (1.5 million), tuberculosis (1.2 million), and malaria (0.6 million). In response, *World Health Organization* adopted Resolution WHA68.15 in 2015, and called for strengthening of emergency and essential surgical care & anesthesia as a component of universal health coverage (UHC) [5]. These two landmark milestones helped reframe surgery as both essential and cost-effective.

However, nearly a decade later, granular data on the availability, sources, and sustainability of funding for surgical care—particularly in low- and middle-income countries—remains scarce. As a result, one of the most fundamental questions in global surgery continues to be insufficiently addressed: *‘who pays for surgical care in the Global South?’* This narrative review examines the financing of surgical services in low and middle-income countries (LMICs), analyzing existing mechanisms, identifying critical gaps and barriers, and exploring potential strategies to advance equitable and sustainable investment in global surgical systems so surgical access is not a privilege, but a universal right.

For this narrative review, we employed a purposive search strategy to ensure broad and relevant coverage of the topic. We searched PubMed, Google Scholar, and the databases of the World Health Organization (WHO) and World Bank for literature published between 2000 and 2025. Both peer-reviewed and grey literature were included, with sources selected based on their relevance to surgical financing models in low- and middle-income countries (LMICs). Editorials, expert commentaries, policy reports, and review articles were also considered to capture diverse perspectives and context-specific insights that inform financing strategies in global surgery.

## The economics of global surgery

Surgical care is often perceived as complex and expensive, yet growing evidence demonstrates that it is not only cost-effective but also economically sustainable [6–8]. Such understanding of the economics of global surgery is essential for its inclusion in health policy and financing priorities, particularly in LMICs.

### The economic burden of surgical disease

Surgically treatable conditions contribute to approximately 30% of the global disease burden [3]. Yet, surgery, with its complex logistics, infrastructure needs, and workforce demands, has struggled to compete for visibility and funding. This gap in access to surgery, not only leads to millions of preventable deaths and disabilities annually but also incurs a significant economic burden on individuals, families, and health systems. While high-income countries (HICs) have largely integrated surgical care into their publicly funded health systems, but LMICs often rely on a fragmented patchwork of out-of-pocket payments, donor funding, and limited government expenditure; resulting in catastrophic health expenditure and impoverishment for many patients [9–13]. *The Lancet Commission on Global Surgery* estimated that the failure to scale up surgical services would lead to an economic loss of $12.3 trillion in LMICs by 2030 due to lost productivity and premature deaths [4]. Strengthening surgical systems also benefits broader health system performance. Investments in surgical capacity improve infrastructure, supply chains, referral systems, and health workforce training—all of which have spillover benefits across health services. Investing in surgery is thus both a health and economic strategy, with long-term benefits for individuals and national development.

### Cost-effectiveness of surgical interventions

Many surgical interventions rank among the most cost-effective public health investments [14]. Basic surgical procedures are on par with widely accepted interventions like oral rehydration therapy or childhood vaccinations if viewed through the lens of cost per disability-adjusted life year (DALY) averted. For example: The cost-effectiveness of individual procedures was: Amputation—$17.66; Emergency caesarean section—$7.42; Elective caesarean section—$20.50; Emergency laparotomy—$8.62; Elective hernia repair—$15.26; Emergency hernia repair—$4.36; Fracture/dislocation reduction—$69.03; Fracture/dislocation fixation—$225.89; Cataract surgery- $50 per DALY averted [15–17].

Moreover, a single operating theatre with basic anesthesia and surgical staff can address multiple conditions—unlike disease-specific vertical programs. This makes surgical infrastructure investment much more impactful.

### Surgery and the sustainable development goals (SDGs)

Surgical care is a critical component in achieving several *Sustainable Development Goals* (SDGs) [18]. For instance, SDG 1 focuses on ending poverty in all its forms; by preventing catastrophic health expenditures through accessible surgical care, families are protected from financial hardship. SDG 3 aims to ensure healthy lives and promote well-being, which includes addressing maternal health, neonatal survival, non-communicable diseases (NCDs), and trauma—all areas where surgical interventions are vital. SDG 8 seeks to promote economic growth and employment; timely surgical treatments can reduce productivity loss from untreated illnesses, thereby contributing to economic stability. Investing in surgical services not only aligns with these development priorities but also accelerates progress by building resilient health systems, promoting equity, and supporting long-term economic growth [19].

### Value-based surgery: A paradigm shift

An emerging concept is that of value-based surgery, which emphasizes delivering high-impact surgical services at the lowest possible cost, while ensuring quality and safety and avoiding surgical care which provides marginal benefits at a disproportionately high cost [20]. In LMICs, several low-cost innovative solutions have demonstrated that cost-effective, high-value surgery is achievable [21–23].

## Current financing mechanisms

Financing for surgical care in LMICs is complex, often inadequate and has historically lacked unified funding efforts [Table 1].

**Table 1:** Comparison of financing models for surgical care in LMICs.

Financing Model	Scalability	Long-term Viability	Population Reach	Common Limitations
Public Sector Funding	Variable, depends on political and fiscal commitment	Can be sustainable with consistent health budgeting	Often focused on urban and tertiary care centers	Limited resource allocation, uneven access in rural areas
Health Insurance Schemes	High if structured for wide enrollment	Durable with strong regulatory frameworks	Potentially broad with inclusive policy design	May exclude informal sector; reimbursement gaps exist
International Aid and Grants	Effective for targeted programs	Typically time-bound or project-specific	Limited to priority areas (e.g., maternal, pediatric)	Lacks continuity; donor priorities may shift
Direct Out-of-Pocket Payment	Readily accessible but inequitable	Financially unstable and burdensome for patients	High usage where insurance is lacking	Can deter care-seeking; leads to economic hardship
Non-profit and Hybrid Models	Promising for localized solutions	Sustainable when paired with internal cross-subsidies	Designed to support underserved groups	Difficult to scale; reliant on innovative governance

### Domestic public financing

In most LMICs, public financing remains the cornerstone of surgical funding [Table 2]. National health budgets support public hospitals, surgical infrastructure, and provider salaries. However, the allocation for surgical services is typically low, often lumped within general hospital expenditures without a dedicated pathway for surgery or anesthesia. Many governments lack the fiscal space or political will to allocate sufficient resources, leading to underfunded surgical infrastructure and workforce shortages.

**Table 2:** Selected regional examples of surgical financing models in LMICs.

Region	Country/Initiative	Financing Approach	Key Features
South Asia	India (AB PM-JAY)	Government-funded national health insurance	Covers 500+ million people; includes surgical services; cashless at point of care
Sub-Saharan Africa	Rwanda and Ghana (Community-Based Health Insurance)	Locally pooled insurance supported by government	Improves access to essential surgical care in rural and informal sectors
Latin America	Regional NSOAP Initiatives with donor support (e.g., in Bolivia, Honduras)	Public-philanthropic partnerships and multilateral aid	Combines strategic planning (NSOAPs) with targeted investments in infrastructure and training

### Insurance-based models

Insurance—both public and community-based—offers a pathway to reduced financial hardship. Examples include *Ayushman Bharat Pradhan Mantri-Jan Aarogya Yojana* (AB PM-JAY) in India which is the largest such scheme in the World and covers over 500 million people, and includes most surgical procedures. It has significantly improved surgical access among low-income populations [24]. Community-Based Health Insurance in countries like Rwanda and Ghana provide essential surgical coverage, often in collaboration with government programs [25]. However, reimbursement rates may be inadequate - deterring private providers and benefits may not percolate down to informal sector workers and rural communities.

### Global assistance

Investment in global surgery is highly cost-effective, and strengthening surgical infrastructure can yield high economic returns [26]. Global assistance for surgery in LMICs has gradually expanded over the past two decades, though it has often relied on mission-based models or single-condition vertical programs rather than systemic investment in surgical systems [27]. One of the most prominent and sustained examples is *Smile Train*, an international nonprofit that has provided funding and support for over 1.5 million cleft lip and palate surgeries in LMICs. Unlike short-term surgical missions, Smile Train emphasizes local capacity-building by training local surgeons, providing infrastructure support, and enabling year-round service delivery—a model that has helped improve continuity and sustainability [28]. Another impactful example comes from targeted pediatric surgical initiatives that address critical gaps in infrastructure. In several African and South Asian countries, philanthropic and multilateral support has enabled the construction and equipping of dedicated pediatric surgery operation theaters within existing government hospitals. Organizations such as *KidsOR* (Kids Operating Room) have worked with local ministries of health to install safe, child-friendly surgical environments, often pairing these efforts with investments in workforce training and postoperative care [29]. Global assistance for cataract surgery in LMICs—through organizations like the *Fred Hollows Foundation*, *Seva*, *Orbis*, and models like *Aravind Eye Care*—has demonstrated how targeted, high-volume, and locally integrated interventions can effectively reduce preventable blindness and strengthen surgical systems. These examples reflect a growing recognition that vertical, procedure-specific interventions—when combined with long-term health system integration—can serve as effective entry points for broader surgical strengthening in resource-constrained settings.

### Out-of-pocket expenditure (OOP)

Even with state funding or insurance, many patients have to bear significant out-of-pocket (OOP) expenses. These costs frequently lead to catastrophic health expenditures (CHE), pushing families into poverty. The cost of surgical care is especially concerning, as it places families at a particularly high risk of financial toxicity [9–13]. Even when surgical treatment is provided free of charge, indirect costs—such as transportation, accommodation, food for patients and caregivers, and lost wages due to time away from work—can still derail a family’s finances, especially when care is accessed far from home. In some regions, over 80% of surgical costs are borne by individuals and families, often leading to debt, asset liquidation, or foregone care.

### Diaspora support and remittances

While not formally tracked, diaspora communities often fund surgeries for family members or support medical missions. These funds supplement local health financing but do not substitute for systemic investment.

## Barriers to adequate financing

Despite evidence that surgery is cost-effective, the finance for surgical care in LMICs remains insufficient and underfunded. The barriers are not merely financial—they are political, structural, informational, and ideological in nature.

### Political will and low visibility

Surgery is often considered ‘luxury’ in global health. Surgeon density is critically low and concentrated in urban centers in majority of LMICs, leaving rural populations underserved. The governments allocate resources to infectious diseases or maternal and child health more readily than surgery. Compounding the issue, global surgery has struggled to align with high-profile frameworks like the *Millennium Development Goals* (MDGs) or *Sustainable Development Goals* (SDGs) monitoring mechanisms, unlike maternal health initiatives that successfully leveraged such platforms for funding and policy traction. This exclusion further diminishes its visibility in national health policy dialogues, perpetuating systemic under-prioritization.

### Lack of data and surgical metrics

The lack of standardized surgical data in LMICs critically undermines effective health system financing and planning. Many countries lack robust health information systems—such as hospital-based registries or electronic medical records—to accurately capture surgical volume, outcomes, and needs. These result in fragmented or incomplete datasets, making it difficult to assess service gaps, monitor interventions, or advocate for targeted investments [4]. The absence of universally accepted surgical metrics further hampers cross-country comparisons and longitudinal tracking, limiting the ability to evaluate the effectiveness of surgical interventions or demonstrate impact. Policymakers and funding bodies rely on high-quality evidence to inform resource allocation, and these persistent data gaps weaken the case for advocating and scaling up surgical care in resource-limited settings [14].

### Fragmentation of efforts and systems

Health partnerships have been a part of global health for many years, but have only recently become a significant part of global surgery efforts. Global surgery efforts have been fragmented because the tendency towards horizontal interventions focused on building health systems and infrastructure, as opposed to vertical interventions focused on specific diseases, has increased more in recent years [30]. Thus, global surgery efforts are often individually initiated, planned, and carried out. Several organizations/ teaching institutes with large footprints in global surgery are working towards making surgery a global health priority; however, there is minimal coordination between groups. In the absence of strategic coordination, these well-intentioned but often overlapping initiatives risk duplicating efforts, diluting impact, and missing opportunities for collective progress—underscoring the urgent need for unified frameworks, shared metrics, and collaborative leadership in global surgery.

### Competing health priorities

LMICs face multiple pressing health challenges, from infectious disease outbreaks and malnutrition to non-communicable diseases and mental health. In this crowded landscape, surgery often struggles to justify its fiscal claim—particularly when outcomes are perceived to be complex and long-term, and costs appear immediate. Additionally, donors often prioritize measurable, disease-specific interventions with immediate results, leading to chronic underinvestment in surgical infrastructure, training, and systems, which require long-term commitments [31].

### Perceived high costs and misconceptions

A persistent misconception is that surgical care is prohibitively expensive and requires high-tech infrastructure. This view fails to consider the cost-effectiveness of basic surgical procedures and the feasibility of delivering safe surgery in resource-constrained settings using task-sharing, modular infrastructure, and frugal innovations [32,33].

## Opportunities and future directions

While the financing of surgical care in LMICs faces formidable challenges, it also presents significant opportunities for innovation, integration, and impact. Recent global health momentum and strategic frameworks like *National Surgical, Obstetric, and Anesthesia Plans* (NSOAPs) are converging to create a new window of opportunity [19]. Such planning is at varying stages of development in different LMICs, offering valuable opportunities for countries for regional partnerships and to share experiences and learn from one another [34–38]. Aligning bilateral donor strategies with country-specific NSOAPs offers a practical way to strengthen health systems. This approach avoids duplication, supports national priorities, and promotes sustainable investments in infrastructure, workforce training, and essential equipment. Multilateral organizations like the WHO, World Bank, and regional development banks have started to recognize the need for integrated surgical care within universal health coverage frameworks. The World Bank’s *Disease Control Priorities* (DCP3) has emphasized surgery as a high-priority investment. Multilateral support can facilitate pooled funding mechanisms by harmonizing donor efforts, provide technical expertise for NSOAP development, and offer catalytic funding to implement and monitor surgical programs.

There is a growing trend toward strategic philanthropy aimed at surgical systems strengthening. Foundations like the *Gates Foundation* and *ELMA Philanthropies* have begun to invest in scalable surgical training and infrastructure. There remains significant untapped potential for philanthropic organizations to support innovation, research, and the implementation of context-specific surgical models in LMICs. Coordinated partnerships between philanthropists and governments can ensure these investments are sustainable and responsive to local needs.

Measures to reduce out-of-pocket expenditures have benefited all countries and income groups, but the gains have been uneven—underscoring the importance of understanding these disparities to design policies that effectively protect against poverty driven by the need to undergo surgery [11].

Innovative financing models, including public-private partnerships and impact investments, are vital for expanding surgical infrastructure and services [39,40]. Harnessing the potential of health insurance schemes can bring LMICs significantly closer to achieving universal access to surgical care [41]. Public-Private Partnerships between public and private sectors can mobilize additional resources, increase efficiency, and expand access to surgical care. Models such as contracting private hospitals for public sector procedures, offering tax incentives for surgical investments, and involving private actors in supply chain management have shown promise. Regulation and equitable service provision must be ensured to avoid exacerbating disparities.

Traditional donor-recipient financing paradigms are increasingly being challenged by innovative models like results-based financing, impact bonds, and blended finance. These approaches aim to improve accountability and leverage private capital for public health goals. For surgical care, such models can support infrastructure development, technology adoption, and workforce capacity building. However, evidence on effectiveness is still emerging, and frameworks must be adapted to the complexities of surgical service delivery.

Some social enterprise hospitals like India’s *Aravind Eye Care* and *Narayana Health* use cross-subsidization in tiered pricing or private service income to subsidize care for the poor [42,43]. Performance-based international aid, linked to measurable outcomes like surgical volume and perioperative mortality rates, can incentivize improvements in surgical care delivery.

Strengthening domestic resource mobilization is critical to achieving universal access to surgical care. LMICs can expand fiscal space through improved tax collection, reallocating inefficient expenditures, and implementing earmarked taxes for health. Prioritizing surgical care in national health budgets and integrating it within insurance schemes can foster sustainability. Strong political will and advocacy are essential to ensure that surgery is not sidelined during national health financing decisions.

Across LMICs, there is a critical gap in research on economics and financing (data on spending, resource allocation, systematic tracking of surgical investments and outcomes), infrastructure, and surgical leadership needed to inform policy. Renewed efforts must focus on equipping countries with the tools for evidence-based decision-making to drive investment in high-quality surgical care [10,44].

Collectively, these strategies underscore the importance of a multifaceted approach to integrate and strengthen surgical services within Universal Health Coverage in LMICs [45]. A paradigm shift in how surgery is viewed within health and development agendas is the key for the financing of global surgery in public health care. Viewing the surgical care as human-right is essential for achieving the *Sustainable Development Goals* and universal health coverage, ensuring equitable financing through rights-based national and health system policies [46].

Resource optimization is essential to making surgical systems financially sustainable. Cost-saving strategies include task sharing, day-case surgeries, use of reusable instruments, local procurement of supplies, and tele-mentoring for remote surgeons. Standardizing care pathways, implementing checklists, and focusing on preventive surgery (e.g., elective hernia repair) can also reduce complications and costs. These efficiency models should be incorporated into national surgical plans to maximize impact. Last but not the least Frugal Surgical Innovations are the key to overcoming the barriers between resource constraints and healthcare quality [47,48].

To move from dependence to sustainability in surgical financing, a paradigm shift is essential—from externally driven, fragmented efforts to integrated, locally sustainable systems. This includes strengthening domestic resource mobilization through improved tax policies and health budget prioritization, expanding regulated public-private partnerships, and incorporating surgical care within national insurance schemes. Emphasis should also be placed on value-based surgery and frugal innovations that maximize impact with limited resources. Furthermore, sustainability can be enhanced through performance-linked donor funding, strategic philanthropic investments, and regional collaborations under National Surgical, Obstetric, and Anesthesia Plans (NSOAPs), which promote shared learning and long-term system strengthening.

## Weakness and strengths of this narrative review

A narrative review such as this one carries some inherent limitations. The absence of a structured and transparent methodology for literature selection increases the risk of subjective interpretation, selection bias, and confirmation bias. Without a systematic search strategy, some relevant publications may be overlooked, leading to incomplete coverage of the evidence base. Moreover, the lack of standardized quality appraisal tools for included sources makes it difficult to assess the robustness or comparability of the data. Narrative reviews typically do not provide a quantitative synthesis or meta-analysis, which limits the ability to evaluate effect sizes or variability across interventions.

Despite these constraints, our narrative review offers several advantages, particularly for a complex topic such as surgical financing in global health. Its flexibility in scope allows for a broad, integrative exploration of issues across diverse stakeholders—governments, donors, multilateral agencies, and philanthropic actors—without being limited by rigid inclusion criteria. This approach facilitates contextualization of evidence within historical, geopolitical, and economic frameworks, drawing from both peer-reviewed studies and grey literature that systematic reviews often exclude. This allowed us to develop and highlight policy-relevant insights that might not emerge from more rigid methodological frameworks. Particularly in a field like global surgery financing—where data are heterogeneous, scattered across disciplines, and sometimes scarce—a narrative review can offer a nuanced understanding that is both informative and action-oriented.

## Conclusion

Surgical care is essential, cost-effective, and transformative. Yet its financing remains one of the most neglected challenges in global health. Ensuring access to safe and affordable surgical services for all will require more than sporadic charity or fragmented funding. It demands a bold and sustained commitment from national governments, global donors, and communities themselves. The question of “*Who Pays for Surgical care in the Global South?*” must be answered not with uncertainty or inertia, but with a shared responsibility anchored in justice, equity, and the recognition that surgical access is not a luxury or a privilege —it is a universal human right.
